# Cytoreductive Surgery and Hyperthermic Intraperitoneal Chemotherapy for Peritoneal Mesothelioma: Outcomes From a Tertiary Cancer Care Center in India

**DOI:** 10.7759/cureus.73658

**Published:** 2024-11-14

**Authors:** Mukurdipi Ray, Bhawani Pathak, Ravi Venugopal, Shwetal Sonvane

**Affiliations:** 1 Surgical Oncology, All India Institute of Medical Sciences, New Delhi, Delhi, IND

**Keywords:** cisplatin, cytoreductive surgery (crs), doxorubicin, hyperthermic perioperative chemotherapy (hipec), ifosfamide, malignant peritoneal mesothelioma, paclitaxel

## Abstract

Background: Malignant peritoneal mesothelioma (MPM) is a rare and aggressive form of cancer arising from the peritoneum. The prognosis for MPM has historically been poor, and treatment options are limited. This study evaluated the impact of cytoreductive surgery (CRS) combined with hyperthermic intraperitoneal chemotherapy (HIPEC) as a treatment modality for MPM.

Materials and methods: This retrospective analysis included 15 patients diagnosed with MPM between 2012 and 2023 at a tertiary referral cancer care center in North India. Patients underwent CRS followed by HIPEC. The study assessed outcomes based on overall survival (OS) and postoperative morbidity rates.

Results: Demographic analysis revealed a female preponderance (n = 9, 60%) and a majority of younger patients, 80% (n = 12) of whom were younger than the age of 50. The mean peritoneal cancer index (PCI) was 14.0, with 60% (n = 9) of patients having a PCI above the mean. The completeness of cytoreduction (CC) varied, with 40% (n = 6) achieving CC0, 33.33% (n = 5) CC1, and 26.67% (n = 4) CC2. Adjuvant chemotherapy was administered to 60% (n = 9) of the patients. The median follow-up period was 25 months, revealing an overall median survival of 27.0 months, with one- and three-year survival rates of 86.7% and 33.3%, respectively.

Conclusion: CRS combined with HIPEC is a viable and effective treatment option for patients with MPM and offers improved survival rates and an acceptable safety profile. These findings support the integration of this treatment modality into the management plan for select patients with MPM, although optimal management is still evolving.

## Introduction

Malignant peritoneal mesothelioma (MPM) is a rare and aggressive malignancy arising from the peritoneal lining and has a historically poor prognosis. The incidence of this disease is increasing, particularly in industrialized nations, posing significant challenges to oncological care [1]. It primarily affects the pleura (60-70%) and peritoneum (20-30%), with less common involvement of the pericardium (1-2%) and tunica vaginalis (1%) [2]. Although asbestos remains the most recognized etiologic factor, its role in MPM is less definitive than that in pleural mesothelioma, suggesting that other pathogenic mechanisms may contribute to its development [3].

The clinical manifestations of MPM are often nonspecific, leading to late-stage diagnosis and a median survival of merely six to 12 months without intervention [4]. Traditional treatments such as systemic chemotherapy and palliative surgery have shown limited efficacy and have not significantly improved patient survival [5].

Cytoreductive surgery (CRS) and hyperthermic intraperitoneal chemotherapy (HIPEC) have emerged as promising modalities for the management of peritoneal surface malignancies. CRS aims to resect all visible tumor deposits and, combined with the locoregional application of chemotherapy via HIPEC, has shown improved survival outcomes in select patient populations [6-7]. HIPEC utilizes the cytotoxic effects of heat treatment to target residual microscopic disease, which can potentially be enhanced by the synergy of hyperthermia with chemotherapeutic agents [8].

The rationale behind HIPEC lies in its dual mechanism of direct cytotoxicity and heat-induced augmentation of chemotherapeutic effects. Regional delivery allows for higher local drug concentrations, which might be more effective against peritoneal disease than systemic chemotherapy, potentially leading to a reduced systemic toxicity profile [9]. Studies have shown varying results with this approach, but a consistent observation is the prolonged survival of patients with complete cytoreduction (CC-0) and favorable histological subtypes, such as epithelioid mesothelioma [10].

Despite these advancements, the literature on the role of HIPEC in treating peritoneal mesothelioma remains limited and consists primarily of retrospective analyses, small case series, and observational studies [4,9]. Furthermore, assessments of perioperative morbidity and long-term quality of life post-CRS and HIPEC treatment have become subjects of increasing interest, with recent research suggesting an acceptable safety profile and improved patient-reported outcomes [11-12].

This study aimed to expand the literature by providing a comprehensive analysis of outcomes following CRS and HIPEC in the treatment of MPM at our institution.

This article was previously posted to the Research Square preprint server on March 29, 2024.

## Materials and methods

A retrospective analysis of a prospectively maintained computerized database extending from 2012 to 2023 was performed. The study was carried out within the Department of Surgical Oncology at All India Institute of Medical Sciences, a tertiary referral cancer care center in New Delhi, North India. Evaluation of the suitability of CRS and HIPEC was performed during weekly multidisciplinary meetings. The team, consisting of surgical oncologists, medical oncologists, and radiologists, assessed patient performance status, comorbidities, extent of disease on computed tomography (CT) scans, and feasibility of maximal cytoreduction.

Fifteen patients with MPM were included in the observational study. All patients had a histological diagnosis of MPM and provided signed informed consent. All patients underwent CRS and HIPEC as per a standard protocol. The exclusion criteria included poor performance status, inability to perform major surgical interventions based on pre-anesthetic evaluation, and unresectable disease determined during laparotomy. Before treatment, the patients underwent physical examination, blood tests (including full blood count; serum electrolytes, creatinine, liver function test, and tumor marker levels), and imaging tests (oral and intravenous contrast CT scans of the chest, abdomen, and pelvis). Mechanical bowel preparation was performed, and prophylactic antibiotics were administered at the time of incision; this process was repeated every four hours. The patients were placed in the supine or low lithotomy position, and a midline incision was made from the xiphisternum to the pubic symphysis. Tumor deposits were documented using the peritoneal cancer index (PCI), and all visible intraperitoneal tumor deposits were excised with CRS [13]. The completeness of the cytoreduction (CC) score was recorded to document residual disease [14]. After surgery, HIPEC was administered for 90 minutes utilizing cisplatin (50 mg/m^2^) and pemetrexed (500 mg/m^2^) dissolved in two liters of dextrose peritoneal dialysis solution. Pelvic and right paracolic gutter drains were inserted before the closure of the abdomen. The patients were managed in the intensive care unit (ICU) until clinically stable (usually 24-48 hours) and then transferred to the surgical ward. Postoperative complications were graded based on the Clavien‒Dindo classification, and patients received continued follow-up care from medical and surgical oncologists upon discharge [15].

Data analysis was conducted using IBM SPSS Statistics for Windows, version 25.0 (IBM Corp., Armonk, NY). Patient characteristics were described using frequency distributions and descriptive analyses. Survival analysis was performed using the Kaplan‒Meier method, with differences between survival curves assessed using the log-rank test. P < 0.05 indicated statistical significance.

## Results

The demographic analysis highlighted a preponderance of females, accounting for 60% (n = 9) of the patients, while males composed 40% (n = 6) of the patients. The majority of patients in the cohort were younger than 50 years, 80% (n = 12) of whom were younger. The average age of the patients was 40.5 years, with a standard deviation of 14.0 years. Notably, all patients (100%, n = 15) had no history of asbestos exposure, a known risk factor for mesothelioma. Neoadjuvant chemotherapy was administered infrequently and was evident in 13.33% (n = 2) of the patients, with the majority (86.67%, n = 13) not receiving such treatment. Histopathological analysis revealed that the most common subtype was epithelioid, which was found in 66.67% (n = 10) of the patients, followed by the mixed subtype in 26.67% (n = 4) and the sarcomatoid subtype in 6.67% (n = 1) (Table 1).

**Table 1:** Characteristics of patients who underwent cytoreductive surgery and hyperthermic intraperitoneal chemotherapy for peritoneal mesothelioma

Category	Number	Percentage (%)
Gender		
Male	6	40.00%
Female	9	60.00%
Age		
<45 years	8	53.33%
>45 years	7	46.67%
Mean (SD)	40.5 (14.0)	
History of asbestos exposure		
Yes	0	
No	15	100.00%
Neoadjuvant chemotherapy		
Yes	2	13.33%
No	13	86.67%
Histopathological subtypes		
Epitheloid	10	66.67%
Sarcomatoid	1	6.67%
Mixed	4	26.67%
Peritoneal Carcinomatosis Index		
Mean (SD)	14.0 (8.4)	
Low	6	40.00%
High (>mean)	9	60.00%
Completeness of cytoreduction (CC)		
CC0	6	40.00%
CC1	5	33.33%
CC2	4	26.67%
Adjuvant chemotherapy		
Yes	9	60.00%
No	6	40.00%

The PCI, a measure of disease extent, exhibited a mean value of 14.0 (SD = 8.4), with a division in the cohort between those with a PCI below the mean (40%, n = 6) and those above (60%, n = 9). Complete cytoreduction (CC0) was achieved in 40.00% (n = 6) of the patients, 33.33% (n = 5) had minimal residual disease (CC1), and 26.67% (n = 4) had residual disease up to 2.5 cm from 2.5 mm (CC2). HIPEC was the standard treatment for all patients, underscoring its established role in the management protocol (100%, n = 15). In addition, a significant proportion of patients received adjuvant chemotherapy (60%, n = 9).

Total peritonectomy was performed in 11 (73.34%) patients, disease-specific peritonectomy in four (26.67%) patients, and total omentectomy in all patients. Total abdominal hysterectomy with bilateral salpingo-oophorectomy was carried out in seven (46.67%) patients. Low anterior resection and appendectomy were both conducted in one (6.67%) patient. Pelvic lymph node dissection was performed in three (20%) patients.

The mean blood loss during the operative procedures was 577 ml, with a standard deviation (SD) of 590 ml. The mean operative duration was 350 minutes, with a mean operative duration of 168 minutes. The median intensive care unit (ICU) stay for the patient cohort was one day, with a range from one to five days. The median hospital stay was eight days, with a range from five to 17 days. Seven patients experienced no significant postoperative complications.

The postoperative complication profile of our patient cohort post-CRS and HIPEC reflects the inherent risks associated with the aggressive management of MPM. The observed complications ranged from mild, self-limiting conditions such as pancreatitis, nausea, and fever (Grade I) in four (26.67%) patients to more severe events such as sepsis and acute renal failure (Grade II) and even life-threatening complications such as pleural effusion (Grade III) and peritonitis (Grade IV). One (6.67%) patient succumbed to a myocardial infarction postoperatively. These findings are consistent with the literature, where the severity and range of complications post-CRS and HIPEC are well documented, with morbidity rates comparable to those in our study, suggesting that CRS and HIPEC, despite their risks, remain viable treatment options for selected patients with MPM [16-19].

**Figure 1:**
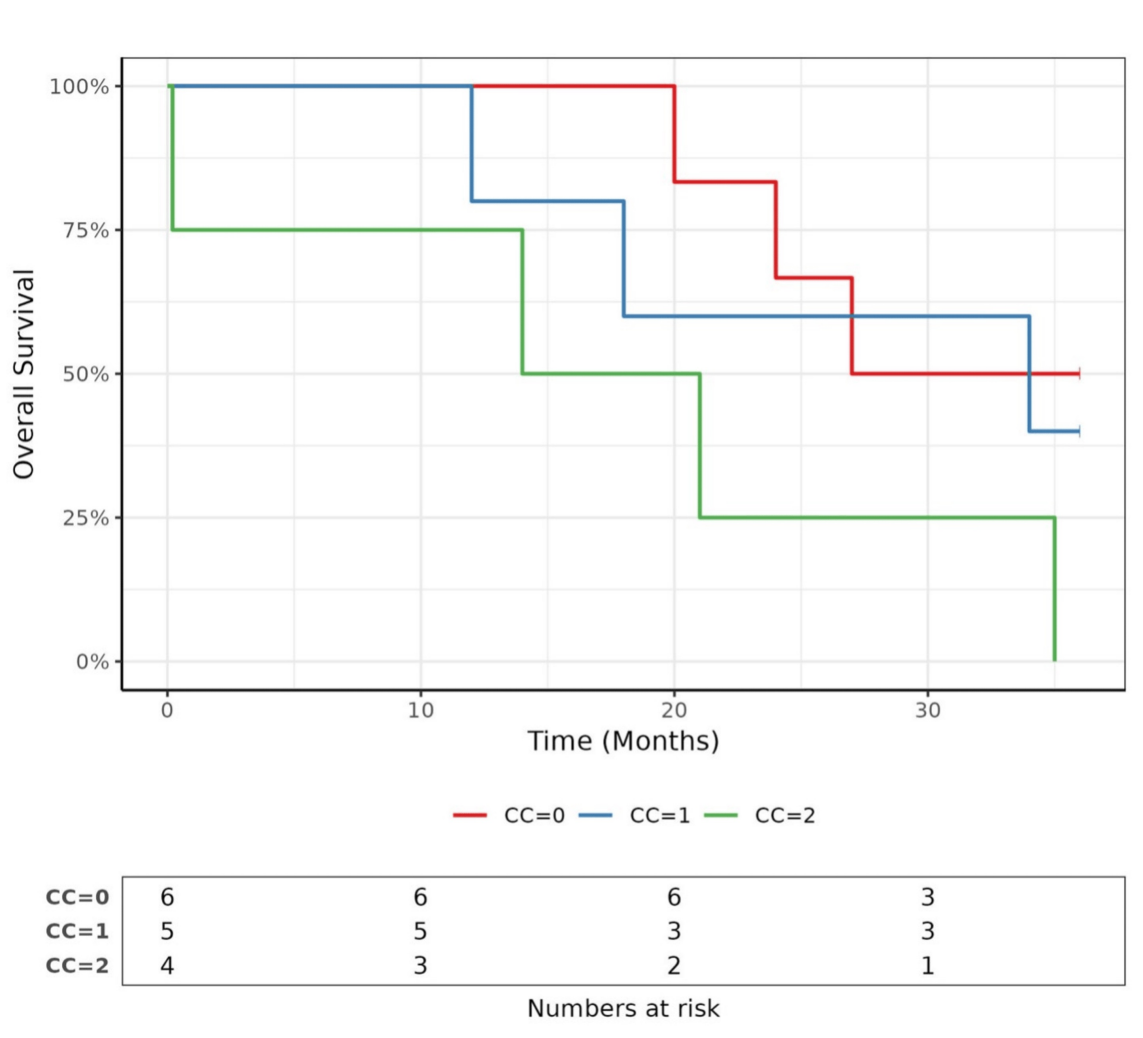
Comparison of survival according to the completeness of cytoreduction

**Figure 2:**
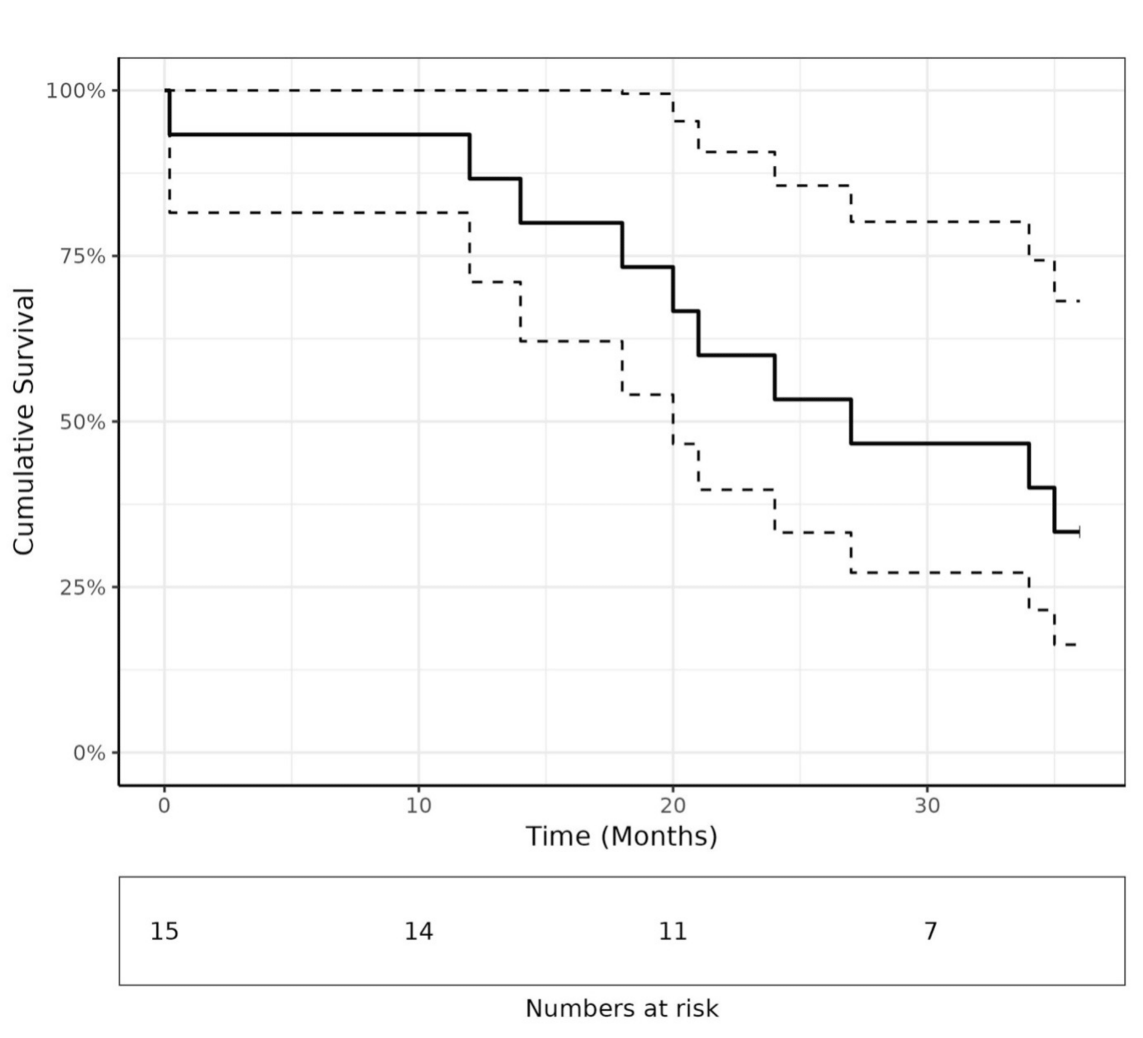
Overall survival of 15 patients treated for peritoneal mesothelioma

The median follow-up period from the time of surgery was 25 months (range 0.2-112 months). The overall median survival of patients with MPM analyzed using the Kaplan‒Meier method was 27.0 months (Figures 1-2).

The one- and three-year survival rates were 86.7% (71.1%-100.0%) and 33.3% (16.3%-68.2%), respectively. According to the univariate analysis, only histological subtype was a significant predictive factor for the overall survival (Table 2). 

**Table 2:** Univariate analysis comparing prognostic variables CC, completeness of cytoreduction; PCI, Peritoneal Carcinomatosis Index; NACT, neoadjuvant chemotherapy

Prognostic variables		P-value
Age	<45 year	0.48
	>45 year	
CC	0	0.18
	1	
	2	
Histology	Epitheloid	0.01
	Sarcomatoid	
	Mixed	
PCI	Low	0.91
	High	
Sex	Male	0.33
	Female	
Adjuvant CT	Yes	0.11
	No	
NACT	Yes	0.1
	No	

## Discussion

Peritoneal mesothelioma, although rare, is anticipated to see an increase in incidence over the next few decades. As a result, it is important to focus on developing treatment strategies tailored to this condition to ensure effective management as the disease presents. Despite attempts with various treatment modalities, including CRS and chemotherapy, the outcomes have been largely unsatisfactory [3].

The demographic distribution within our study, notably a female majority and a prevalence of patients under the age of 50, raises questions about the typical profile of mesothelioma patients and suggests that other environmental or genetic factors may contribute to disease development. The absence of asbestos exposure in our patient cohort is particularly striking and aligns with emerging research suggesting alternative etiological pathways [20,21]. The limited size of the sample could have influenced the outcomes.

HIPEC involves the circulation of heated chemical agents within the peritoneal cavity post-cytoreduction, aiming to eliminate the residual microscopic disease. The rationale for hyperthermia is twofold: it enhances the cytotoxicity of chemotherapeutic agents and facilitates deeper penetration into tissues [22]. Studies have demonstrated that HIPEC combined with complete cytoreduction can lead to median survival rates that significantly exceed those achieved with traditional therapies [23].

Despite these promising developments, the administration of HIPEC remains complex, with considerable debate regarding its indications, optimal timing, and patient selection criteria. The procedure is associated with significant morbidity and requires careful patient evaluation and management by a multidisciplinary team [24].

The administration of neoadjuvant chemotherapy in our study was reserved, and only 13.33% (n = 2) of patients received this treatment. This percentage is comparatively low when juxtaposed with other studies, where neoadjuvant chemotherapy is more commonly used as a part of multimodal treatment [5]. The rationale behind this conservative approach to neoadjuvant chemotherapy in our cohort could be multifaceted and potentially influenced by factors such as the timing of presentation, the burden of disease, and the perceived responsiveness of the tumor to systemic therapy.

In our series, epithelial mesothelioma was the most common histological subtype (66.67%), which is associated with a better prognosis than are the sarcomatoid and mixed subtypes [25]. The distribution of subtypes in our study aligns with the established literature that also reports that epithelioids are the most common subtype of peritoneal mesothelioma. Among our patients, 26.67% (n = 4) had the mixed subtype, followed by the sarcomatoid subtype (n = 1).

When addressing the clinical management outcomes detailed in the provided data, it is essential to consider the PCI and the completeness of cytoreduction (CC), both of which are well-established prognostic indicators for MPM. Our cohort presented a mean PCI of 14.0, which is a pivotal finding considering that a higher PCI is correlated with poorer outcomes, as indicated in studies where CRS combined with HIPEC was evaluated [5]. The fact that 60% of our patients had a PCI above the mean might reflect an advanced disease stage at presentation and could influence survival outcomes, despite the aggressive treatment approach adopted.

Our cohort achieved a CC0 rate of 40% (n = 6), which compares favorably with the published literature. For instance, Sugarbaker and colleagues reported CC0 rates varying from approximately 40-50% in selected patient groups undergoing CRS and HIPEC, confirming the importance of complete cytoreduction in improving outcomes [6]. Other studies have reported CC0 rates ranging between 30% and 60%, with higher rates associated with specialized centers that frequently perform these complex treatments (5, 23%). The CC1 and CC2 rates, representing minimal and more extensive residual disease, respectively, were 5 (33.33%) and 4 (26.67%). These findings underscore the inherent challenges in achieving complete cytoreduction and align with broader clinical experience, where complete macroscopic clearance is not always feasible due to tumor spread and patient factors [26].

We have performed HIPEC in four instances for CC2, aiming to alleviate intractable ascites and enhance the patient's quality of life with their consent. We refer to it as palliative HIPEC, considering that according to the literature, a single HIPEC procedure may decrease significant ascites in up to 90% of cases. The universal application of HIPEC in our cohort reinforces its role as a cornerstone in the current standard of care for MPM, as it has been shown to improve survival in patients who underwent complete CRS [23]. The high rate of adjuvant chemotherapy usage (n = 9, 60%) further exemplifies the aggressive therapeutic strategy employed in our center, although the impact on survival remains to be conclusively determined.

The surgical procedures, ranging from total resection to organ-specific resection and lymph node dissection, reflect the tailored approach to the extent of diseases, aiming to achieve the best possible cytoreductive outcomes. However, the relatively extensive surgeries performed may contribute to variability in postoperative recovery and morbidity, factors that require careful preoperative assessment.

When interpreting the operative and postoperative data of MPM patients in our study, surgical management was considered to be appropriate, as reflected by the mean blood loss and operative duration. The average blood loss of 577 ml, although significant, is within acceptable limits for major abdominal surgeries, and the mean operative time of 350 minutes indicates the complexity and extent of the procedures performed, such as peritonectomy and organ resections. These operative parameters are consistent with those of other specialized centers performing similar extensive cytoreductive surgeries [27].

A median ICU stay of one day and a hospital stay of eight days are indicative of an efficient postoperative care protocol, optimizing patient recovery and resource utilization. These durations are comparable to or better than those reported in larger series, where the complexity of the surgery can lead to longer ICU and hospital stays [5]. The relatively short ICU stay also suggested a high level of surgical and anesthetic expertise, as well as effective postoperative management protocols.

Postoperative complications occur in a pattern that is not uncommon in high-risk abdominal surgeries. The spectrum of complications observed, ranging from mild (grade I) to more severe (grade IV), provides a real-world snapshot of the potential risks associated with aggressive surgical management of MPM. Notably, the incidences of Grade III and IV complications, as well as single-stage mortality, underscore the necessity of careful patient selection and the inherent risks of the disease and its treatment.

The overall median survival of 27.0 months, with one- and three-year survival rates of 86.7% and 33.3%, respectively, offers a meaningful addition to the literature on MPM, which generally reports a median survival ranging from 12 to 27 months (23). On univariate analysis, only histological subtype emerged as a predictive factor for overall survival, while PCI and CC were not significant. This could be attributed to the small sample size of our study.

The limitations of this study include its retrospective nature, reliance on data from a single center, limited sample size, and absence of Ki67 reporting in the histopathological analysis.

## Conclusions

MPM is a rare disease group but represents a unique entity. The standard of care is CRS combined with HIPEC. However, the potential benefit of chemotherapy in the adjuvant or neoadjuvant setting is uncertain. Nevertheless, in specialized centers, extensive surgical procedures involving HIPEC have been observed to improve survival outcomes, as evidenced by our institution's experience, although optimal management is still evolving.
